# Effect of relaxation-dependent adhesion on pre-sliding response of cartilage

**DOI:** 10.1098/rsos.172051

**Published:** 2018-05-23

**Authors:** Guebum Han, Melih Eriten

**Affiliations:** Department of Mechanical Engineering, University of Wisconsin-Madison, Madison, WI 53706, USA

**Keywords:** pre-sliding response, relaxation time, work of adhesion, interfacial failure, stick–slip contrast, cartilage

## Abstract

Possible links between adhesive properties and the pre-sliding (static) friction response of cartilage are not fully understood in the literature. The aims of this study are to investigate the relation between adhesion and relaxation time in articular cartilage, and the effect of relaxation-dependent adhesion on the pre-sliding response of cartilage. Adhesion tests were performed to evaluate the work of adhesion of cartilage at different relaxation times. Friction tests were conducted to identify the pre-sliding friction response of cartilage at relaxation times corresponding to adhesion tests. The pre-sliding friction response of cartilage was systematically linked to the work of adhesion and contact conditions by a slip-based failure model. It was found that the work of adhesion increases with relaxation time. Also, the work of adhesion is linearly correlated to the resistance to slip-based failure. In addition, as the work of adhesion increases, the adhered (stick) area at the moment of failure increases, and the propagation rate of the annular slip (crack) area towards its centre increases. These findings offer a mechanistic explanation of the pre-sliding friction behaviour and stick–slip response of soft hydrated interfaces such as articular cartilage and hydrogels. In addition, the linear correlation between adhesion and threshold to slip-based failure enables estimation of the adhesive strength of such interfaces directly from the pre-sliding friction response (e.g. shear wave elastography).

## 1. Introduction

Articular cartilage is a biological tissue that cushions and lubricates the interfaces between the tips of long bones in mammals. Articular cartilage is composed of two phases: a fluid phase (water and electrolytes, 65–80% of its wet weight) and a solid phase (collagen fibrils, proteoglycans, chondrocytes and other glycoproteins) [1,2]. The interaction of the solid matrix (collagen fibrils and proteoglycans) and the pressurized fluid effectively distributes loads experienced in daily life activities [3,4]. The exceptional lubrication property of cartilage protects it from severe wear and friction. Excellent mechanical and tribological properties of cartilage make it last over the lifetime of most people. These fascinating characteristics of cartilage have inspired researchers to investigate its mechanics and mimic its behaviour with soft hydrated materials [5].

Numerous studies have investigated the tribological characteristics of cartilage in a sliding (kinetic) friction regime. Many lubrication mechanisms such as hydrodynamic [6], boundary [7,8], weeping [9], boosted [10], biphasic self-generating [11], elastohydrodynamic [12] and biphasic boundary [13,14] lubrications have been proposed for articular cartilage. Previous studies reported that the pressurization of interstitial fluid has a pronounced effect on cartilage lubrication [4,15,16]; the pressurized fluid sustains the applied contact load and effectively slides the contact area over the solid matrix with a low friction coefficient. In particular, Caligaris & Ateshian [15] suggested that the coefficient of friction under stationary contact increases with time, while the coefficient of friction under migrating contact stays at a low value for long durations. They explained that the low coefficient under migrating contact is because the contact area migrates faster than the diffusion rate of interstitial fluid, so that the fluid does not have enough time to flow away from the contact, resulting in the sustained pressurization of interstitial fluid under the contact [15]. Also, they showed that the pressurization of interstitial fluid is much more effective than boundary lubrication [15]. Park *et al*. [17] performed friction tests on cartilage using atomic force microscopy (AFM) and showed that the absence of the pressurized fluid due to a small contact area increases the coefficient of friction.

While tribological properties of cartilage in the sliding (kinetic) friction regime have been extensively studied, the pre-sliding (static) friction response of cartilage has drawn less attention. Although a few studies reported that the static friction coefficient of cartilage increases with an increase in the contact dwelling duration before sliding [13,18,19], the underlying mechanisms and mechanics of the pre-sliding response were not addressed. A previous study on the wear of articular cartilage in sliding conditions showed that cartilage exhibits severe wear under a stick (static)–slip (kinetic) friction regime rather than under a sliding (kinetic) friction regime [20]. Also, articular cartilage often encounters pre-sliding friction during daily joint movements when joint movements change from a static state to a dynamic state. This evidence suggests that a systematic understanding of the pre-sliding (stick, static) regime is needed to understand wear damage and fill the gaps in our knowledge. A recent paper found that the pre-sliding friction response of hydrogels is significantly affected by poro-elasticity-driven adhesion [21]. Hydrogels and cartilage have similarities in that their matrices are surrounded by fluid, which inspired us to investigate if the work of adhesion is a parameter governing the pre-sliding friction response of cartilage. To the best of our knowledge, the pre-sliding friction response of cartilage itself is not well known, and the effect of relaxation-driven adhesion on the pre-sliding friction response of cartilage has not been studied in the literature.

The pre-sliding friction response of cartilage can be systematically estimated by using a mechanics-based model. As various studies on the pre-sliding friction responses of materials have shown, model-based analysis provides a better understanding of mechanisms [22,23]. In this study, we will employ a mechanics-based model to link relaxation-driven adhesion to the pre-sliding friction response of cartilage. The model we use is a hybrid model previously applied to elastomers and gels. In particular, the stick-to-slip transitions in the pre-sliding response are modelled after Savkoor's model, which was originally proposed and applied to elastomers [24,25]. Unloading of the contact after slip-based failure is modelled after the ‘naive modelling’ approach of Brochard-Wyart & de Gennes [26]. That model was originally employed to explain the stick–slip processes observed in soft materials. Savkoor's model is useful to account for adhesive interactions during the pre-sliding response. The model proposed by Brochard-Wyart and de Gennes helps to explain the stable failure propagation over the contact and transitioning to fully sliding. A combination of those two models is referred to as the ‘slip-based failure model’ in this paper.

The aims of this study are to investigate the relation between adhesion and relaxation time in articular cartilage, and to investigate the effect of relaxation-dependent adhesion on the pre-sliding friction response of cartilage. Adhesion tests were performed to evaluate the work of adhesion of cartilage at different relaxation times. Friction tests were conducted to identify the pre-sliding friction response of cartilage at relaxation times corresponding to adhesion tests. Finally, the experimental pre-sliding friction response of cartilage was systematically correlated to the slip-based failure model to establish a quantitative correlation between the work of adhesion and the resistance of cartilage to frictional loading. Section 2 explains the experimental methods and the slip-based failure model. The experimental methods include sample preparation, adhesion tests and pre-sliding friction tests. A brief overview of a slip-based failure model is provided, and the process of applying the model to experimental pre-sliding data is described. Section 3 lists the results of adhesion and pre-sliding friction tests, and the results of fitting the slip-based failure model to the pre-sliding friction response. Section 4 discusses the physical meaning of the results, followed by conclusions in §5.

## 2. Material and methods

### 2.1. Sample preparation

Three full-thickness cartilage samples (*N* = 3) were harvested from the patellae of three porcine joints (5–6 months old). Cylindrical samples were obtained with a 6 mm diameter coring tool and a scalpel, and their subchondral bones were trimmed using a microtome. The back surface of each sample near the deep zone was attached to a Petri dish using an instant adhesive (Super Bonder 495; Loctite, Ontario, Canada). Samples were kept hydrated during preparation in Dulbecco's phosphate-buffered saline (DPBS) with protease inhibitors, and allowed to relax for about an hour to reach equilibrium before testing. Mechanical testing was performed at five different locations (*n* = 5) on each of the three cartilage samples (*N* = 3). The average and standard deviation of material properties were calculated based on *Nn* = 15.

### 2.2. Adhesion test

**Figure 1.**
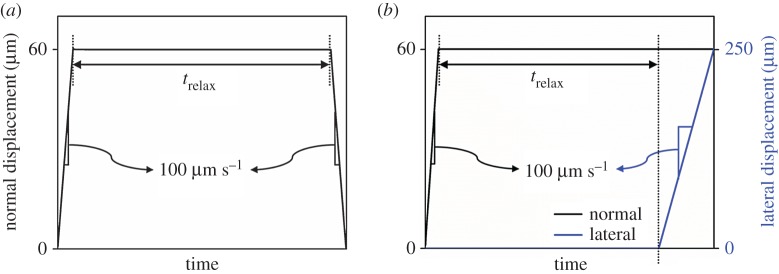
(*a*) Normal displacement profile of adhesion tests. (*b*) Normal and lateral displacement profiles of friction tests. All tests were conducted under displacement control. The profiles were plotted by using one of the experimental results, showing the displacements were controlled well.

Adhesion tests on the articular surface of hydrated cartilage were performed to investigate the effect of relaxation time on the work of adhesion. Tests were conducted on a Bruker TI950 TriboIndenter (Hysitron Inc., MN) using a sapphire spherical indenter with a radius of 1 mm. A constant normal displacement of 60 µm was instantaneously applied with a velocity of 100 µm s^−1^, held until a set relaxation time and removed with a velocity of 100 µm s^−1^ (figure 1*a*). At this constant displacement, the contact area was small compared with the probe radius, and thus elastic (Hertzian) contact assumptions prevailed. The relaxation (hold) time, *t*_relax_, was an independent variable, and 0, 5, 10, 20, 50, 100 and 200 s were used. The pull-off force, *F*_pull-off_, was defined as the maximum negative force of a load–displacement curve. The work of adhesion was calculated through two different methods. The first method was based on the Johnson–Kendall–Roberts (JKR) contact model [27], and the work of adhesion is estimated from measured values of *F*_pull-off_,

2.1 $$\gamma =-\frac{F_{\mathrm{pull}\text{-}\mathrm{off}}}{1.5\pi R},$$

where *R* is the radius of the indenter. The average and standard deviation of the work of adhesion per each relaxation time were calculated with 15 measured values (*Nn* = 15). The second method was based on the normalized relaxation curve, where *γ_i_* and *γ_f_* are the work of adhesion at the unrelaxed and fully relaxed states, as in [21],

2.2 $$\gamma (t)=\gamma_{f}-(\gamma_{f}-\gamma_{i})\left(\frac{F_{n}(t)-F_{n,f}(t)}{F_{n,i}(t)-F_{n,f}(t)}\right),$$

where *F_n_*_,*i*_(*t*), *F_n_*_,*f*_(*t*) and *F_n_*(*t*) are the normal loads at the initial (*t*_relax_ = 0 s) and final (*t*_relax_ = 200 s) relaxation curve and arbitrary time, respectively. Here, *γ_i_* and *γ_f_* were the work of adhesion at *t*_relax_ = 0 s and *t*_relax_ = 200 s from equation (2.1), respectively. The normalized relaxation curve was obtained by taking the average of 15 relaxation curves from *t*_relax_ = 200 s. Eventually, the measured (equation (2.1)) and predicted (equation (2.2)) adhesion were compared to check which one can suitably explain the pre-sliding friction response of cartilage. A previous study explained the pre-sliding friction response of hydrogels with relaxation curve-based adhesion [21].

### 2.3. Friction tests

Friction tests were performed to examine the influence of relaxation-dependent adhesion on the pre-sliding friction response of articular cartilage. The same instrument and indenter used in the adhesion tests were used for the friction tests. A normal displacement of 60 µm was applied with a velocity of 100 µm s^−1^ and maintained until the end of each test. A tangential displacement of 250 µm at a sliding velocity of 100 µm s^−1^ was applied after set relaxation (hold) times, *t*_relax_, that corresponded to the relaxation times used in the adhesion tests. The relatively slow tangential sliding was chosen intentionally to ensure that frictional interactions stem from elastic deformation, and not from hydrated lubrication [28,29]. The normal and tangential displacement profiles are shown in figure 1*b*. Reported values from the friction tests were calculated with 15 measured values (*Nn* = 15).

### 2.4. Slip-based interfacial failure model

#### 2.4.1. Brief overview of the slip-based failure model

**Figure 2.**
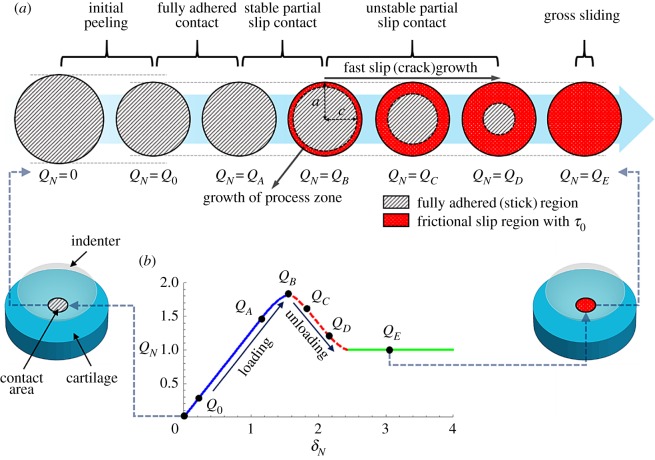
(*a*) Evolution of the stick (dashed-grey) and slip zones (dotted-red) over the projected contact area during tangential loading. (*b*) Normalized tangential force versus displacement with critical points (*Q*_O_: end of initial peeling; *Q*_*A*_: onset of stable partial slip (failure); *Q*_*B*_: onset of unstable partial slip; *Q*_*C*_: rapid slip (crack-like failure) growth, *Q*_*D*_: moment before the onset of gross sliding; *Q*_*E*_: gross sliding). The shear traction distribution over the contact radius at points *Q*_*A*_, *Q*_*B*_, *Q*_*C*_ and *Q*_*D*_ is shown in the electronic supplementary material, figure S1.

The slip-based failure model addresses fracture-like failure induced in a spherical contact under combined normal and tangential loading, *Q*, and adhesive interactions (figure 2). First, normal loading creates a circular contact area that is larger than the Hertzian contact area due to adhesion, illustrated as *Q_N_* = 0. A quick peeling occurs upon application of a tangential force, *Q_N_* = *Q*_0_, and the contact area reduces to the Hertzian contact area to balance out the applied normal load, referred to as initial peeling. After that point, the contact area remains fully adhered until a critical load, *Q_N_* = *Q_A_*, referred to as fully adhered contact. As *Q_N_* increases from *Q_A_* to *Q_B_*, the singular tangential tractions at the edge of that contact area yield a critical stress intensity factor, and debonding slightly occurs towards the centre of the contact area. The debonded portion of the contact area undergoes frictional slip with constant tangential traction, *τ*_0_, while the central portion of the contact area remains fully adhered, referred to as stable partial slip contact. *τ*_0_ is the residual strength of the physically and/or chemically adsorbed layers at the interface, and is assumed to be independent of normal tractions (see [30,31] for experimental evidence). After the critical point where *Q_N_* becomes maximum, *Q_N_* = *Q_B_*, the frictional slip zone grows quickly and unstably towards the centre of the contact area, referred to as unstable partial slip contact. Gross sliding eventually commences after the frictional slip zone extends over the whole contact area, illustrated as *Q_N_* = *Q_E_*. We employ stable growth of the slip zone size to analyse displacement-controlled experiments. The following sections (§§2.4.2–2.4.4) introduce how each stage of the model is mathematically expressed in a normalized form, which is directly used to fit the experimental pre-sliding friction response. The electronic supplementary material consists of the derivation of the normalized forms. Section 2.4.5 provides the application process of the model to the experimental results with a schematic diagram.

#### 2.4.2. Fully adhered contact (*Q*_0_ – *Q*_*A*_)

Under fully adhered contact (figure 2), the tangential displacement, *δ*, is linked to *Q* linearly as follows:

2.3 $$\delta =\frac{\lambda Q}{8a};\;\lambda =\frac{2-\nu_{1}}{G_{1}}+\frac{2-\nu_{2}}{G_{2}},$$

where *a* is the contact radius of the fully adhered contact, and *ν_i_* and *G_i_* are Poisson's ratio and the shear modulus of the *i*th contacting material. The normalized form of the tangential force, *Q_N_*, and the tangential displacement, *δ_N_*, are defined as follows:

2.4 $$Q_{N}=\frac{Q}{Q_{S}}=\frac{Q}{\tau_{0}\pi a^{2}}$$

and

2.5 $$\delta_{N}=\frac{2\delta }{a\lambda \tau_{0}},$$

where *Q_S_* = *τ*_0_*πa*^2^ is the tangential force when the whole of the contact area slips (gross sliding). Then the relationship between *Q_N_* and *δ_N_* in the fully adhered contact is established by substituting equation (2.4) and equation (2.5) into equation (2.3) as follows:

2.6 $$Q_{N}=\frac{4}{\pi }\delta_{N}.$$

#### 2.4.3. Stable partial slip (*Q*_*A*_ – *Q*_*B*_)

After the onset of the stable partial slip (*Q_N_* = *Q_A_*) (figure 2), the normalized forms of the tangential force, *Q_N_*, and the tangential displacement, *δ_N_*, are defined as follows:

2.7 $$Q_{N}=\frac{Q}{Q_{S}}=\frac{Q}{\tau_{0}\pi a^{2}}=\Theta \chi^{3/2}+\frac{2}{\pi }\left(\chi \sqrt{1-\chi^{2}}+\text{co}{\text{s}}^{-1}\chi \right)\;$$

and

2.8 $$\delta_{N}=\frac{2\delta }{\lambda \tau_{0}a}=\frac{\pi }{4}\Theta \sqrt{\chi }+\sqrt{1-\chi^{2}},$$

where $\Theta =Q_{A}/Q_{S}$ is the ratio of the critical to sliding tangential forces (referred to as stick–slip contrast) and χ=c/a is the normalized radius of the fully adhered (stick) contact area (figure 1).

#### 2.4.4. Unstable partial slip (*Q*_*B*_—before gross sliding)

In the regime of the unstable partial slip (figure 2), to meet the constraints in a set-up with displacement control, the relative tangential velocity of the contacting interfaces, $\dot{\delta }$, is assumed to scale linearly with the extension rate of the annual frictional slip zone, $\;{\dot{\chi }}_{\mathrm{un}}$. Then its normalized form, ${\dot{\delta }}_{N}$, can be expressed as follows:

2.9 $${\dot{\delta }}_{N}=-\kappa {\dot{\chi }}_{\mathrm{un}},$$

where *κ* is a scaling constant. The normalized tangential displacement during the unstable partial slip, *δ_N_*, can be eventually expressed as follows:

2.10 $$\delta_{N}=\delta_{N}^{B}+\kappa (\chi_{c}-\chi_{\mathrm{un}}),$$

where $\delta_{N}^{B}$ is the normalized tangential displacement at *Q_N_* = *Q_B_*, *χ*_c_ is the normalized radius of the fully adhered (stick) contact area at *Q_N_* = *Q_B_* (see equation (S20), electronic supplementary material) and $\chi_{\mathrm{un}}=C_{\mathrm{un}}/a$ is the normalized radius of the stick contact area during unloading. The normalized tangential force, *Q_N_*, is assumed to attain the same value corresponding to the stable partial slip (equation (2.7)) with *χ*_un_ instead of *χ*. Note that kinematic constraints localized at the interface might exhibit nonlinear correlation to the crack-tip velocities. The linear correlation offered here should be treated as a first-order approximation. In addition, a similar linear relation between the imposed displacement rates and crack-tip velocities is used by Brochard-Wyart and de Gennes to explain the linear decohesion observed experimentally on soft PDMS-on-glass contacts [32]. The physical arguments behind this linear correlation will be revisited in the Discussion.

#### 2.4.5. Application of the slip-based failure model

**Figure 3.**
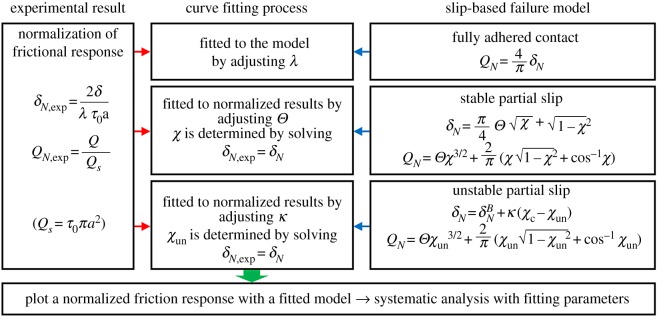
Schematic diagram of applying the slip-based failure model to pre-sliding friction results. In the stable and unstable partial slips, *Q_N_* is calculated by substituting *χ* and *χ*_un_ into the corresponding equations above. *χ*_c_ is determined by using equation (S20) in the electronic supplementary material.

This section briefly explains the application of the model to the experimental results with a schematic diagram in figure 3. The results of pre-sliding friction tests are re-plotted as normalized forms, *δ_N_*_,exp_ − *Q_N_*_,exp_ ( figure 3). The initial portion of the normalized friction result is fitted to the model (fully adhered contact) by adjusting the parameter *λ* (equation (2.6)). The following portion of the normalized friction result is fitted to the model (stable partial slip contact) by adjusting the parameter *Θ*, determining *χ* from *δ_N_*_,exp_ = *δ_N_*, and calculating *Q_N_* from the determined *χ* (equation (2.7)). The unloading portion of the normalized friction result is fitted to the model (unstable partial slip) by adjusting a fitting parameter of *κ*, determining *χ*_un_ from *δ_N_*_,exp_ = *δ_N_* and calculating *Q_N_* from the determined *χ*_un_ (equation (2.7)). The effect of parameters *Θ* and *κ* on the model is illustrated in the electronic supplementary material, figure S2.

## 3. Results

### 3.1. Work of adhesion of cartilage

**Figure 4.**
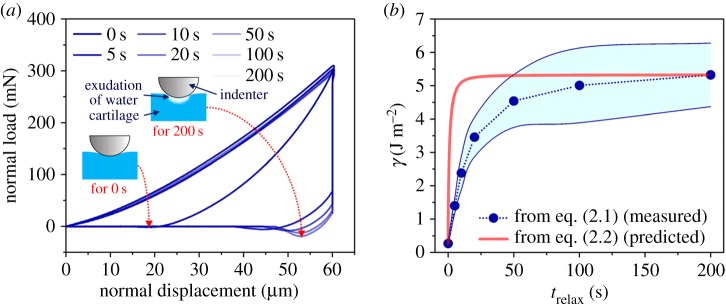
(*a*) Representative results of adhesion tests; (*b*) work of adhesion as a function of relaxation time. The work of adhesion from equation (2.1) is obtained based on the measured values from the adhesion tests. The work of adhesion from equation (2.2) is predicted based on the normalized relaxation curve. The standard deviations were connected using modified Bezier curves.

The relaxation (hold) time had a pronounced effect on the work of adhesion of cartilage. Figure 4*a* shows the representative results of the adhesion tests at different relaxation times. The pull-off force increased with an increase in relaxation time. The measured pull-off force is converted to the work of adhesion through equation (2.1) (figure 4*b*). The work of adhesion increased quickly at the beginning of the relaxation process and gradually settled to a steady value after *t*_relax_ = 50 s. The minimum work of adhesion was 0.27 ± 0.07 J m^−2^ at *t*_relax_ = 0 s. The maximum work of adhesion was 5.33 ± 0.96 J m^−2^ at *t*_relax_ = 200 s, which was about 20 times greater than the value at *t*_relax_ = 0.

In addition to the directly measured work of adhesion, the work of adhesion as estimated by the normalized relaxation curve with *γ_i_* and *γ_f_* is also shown in figure 4*b*. The normalized relaxation curve for that estimation was taken from the average of 15 relaxation responses at *t*_relax_ = 200 s. *γ_i_* and *γ_f_* were from the measured work of adhesion at *t*_relax_ = 0 s (0.27 ± 0.07 J m^−2^) and *t*_relax_ = 200 s (5.33 ± 0.96 J m^−2^), respectively. Finally, the predicted work of adhesion was obtained by substituting these values into equation (2.2) (figure 4*b*). Although the measured and predicted adhesion results had similar trends, they were not in complete agreement.

### 3.2. Pre-sliding friction response of cartilage

**Figure 5.**
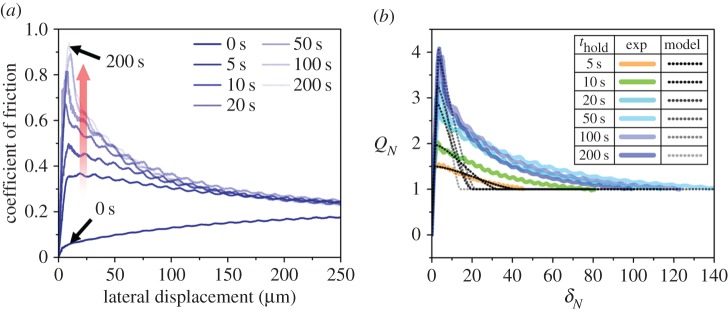
(*a*) Representative results of pre-sliding friction tests; (*b*) curve fitting of friction tests.

The pre-sliding friction response of cartilage strongly depended on the relaxation (hold) time, as did the work of adhesion. The ratios of the tangential to normal loads, measured on cartilage after predefined relaxation times, are presented in figure 5*a* as a function of the tangential displacement. This ratio is referred to as the coefficient of friction in classical terms. Note that, except for *t*_relax_ = 0 s, a normal load during friction tests does not change significantly, and thus the ratio shown resembles a tangential load. As the relaxation time increased, the pre-sliding friction increased to a peak value, and then gradually decreased and transitioned to sliding friction values. As for *t*_relax_ = 200 s, the coefficient of friction at the peak (0.93 ± 0.07) was four times larger than the coefficient of friction (0.25 ± 0.02) in the sliding regime. By contrast, when the relaxation time was relatively short (e.g. *t*_relax_ = 0 s), the pre-sliding friction response showed a smooth transition to a gross sliding regime.

### 3.3. Application of the slip-based failure model

The results of the pre-sliding friction tests were normalized, and then the normalized results were fitted to the slip-based failure model (figures 3 and 5*b*). Specifically, the loading, peak friction and unloading portions of the pre-sliding friction response were fitted to the model by adjusting fitting parameters (figure 3). The results from *t*_relax_ = 0 s were omitted from the fitting because the friction response did not show the peak friction. Representative normalized experimental curves fitted to the slip-based models are shown in figure 5*b*. In the case of *t*_relax_ = 5 s, the slip-based failure model predicted the overall pre-sliding friction response from the loading portion to the unloading portion. However, as the relaxation time increased, the model significantly deviated in the extended unloading portion (about 20 < *δ_N_*) even though it still well simulated the loading, peak friction and initial unloading portions of the pre-sliding friction response.

**Figure 6.**
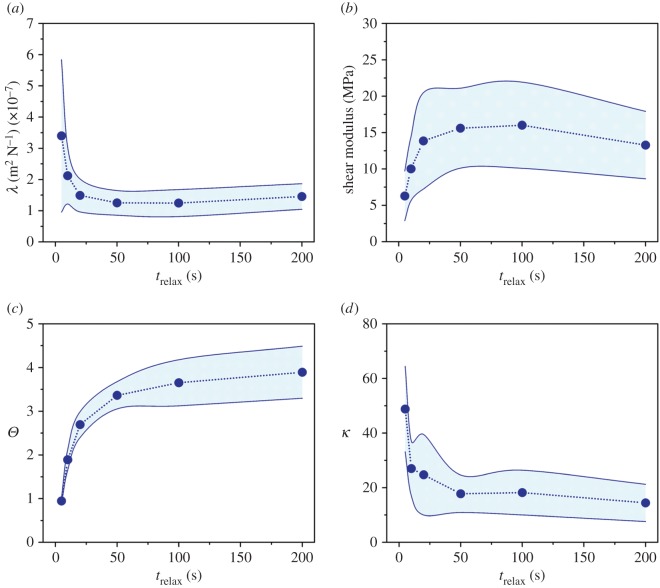
Results of (*a,c*,*d*) fitting parameters and (*b*) shear modulus. Shear moduli were calculated by substituting the determined *λ* into equation (2.3), and Poisson's ratio of 0.25 was used. The standard deviations were connected using modified Bezier curves.

The linear loading portion of the pre-sliding friction response was influenced by the relaxation time, and the influence was well captured by the model (fully adhered contact, figure 3) with a properly selected value of *λ*. *λ* rapidly decreased in a relatively short relaxation time (*t*_relax_ < 20 s) and became stable (*t*_relax_ > 20 s) (figure 6*a*). Shear moduli were calculated based on the determined *λ* (figure 6*b*). The shear modulus showed an increasing trend as the relaxation time increased. It ranged from 6.29 ± 3.01 MPa at *t*_relax_ = 5 s to 13.28 ± 4.63 MPa at *t*_relax_ = 200 s (16.01 ± 5.92 MPa at *t*_relax_ = 100 s). These results were consistent with the moduli calculated using the load–displacement curves measured in the adhesion tests (see the electronic supplementary material).

The degree of peak friction and the critical adhered (stick) area depended on the relaxation time, and they were quantitatively evaluated by using the model (stable partial slip; figure 3) with related parameters. The value of the peak friction, also called the stick–slip contrast, was quantified by a fitting parameter of *Θ*. *Θ* rapidly increased in a relatively short relaxation time (*t*_relax_ < 50 s) and gradually reached a plateau (*t*_relax_ > 50 s) (figure 6*c*). The minimum value was 0.95 ± 0.07 at *t*_relax_ = 5 s, and the maximum value was 3.89 ± 0.59 at *t*_relax_ = 200 s. The normalized radius of the critical adhered area, *χ*_c_, was estimated by substituting the determined *Θ* into equation (S20) (see the electronic supplementary material). *χ*_c_ increased from 0.8 at *t*_relax_ = 5 s to 0.98 at *t*_relax_ = 200 s. The relationship between *Θ* and *χ*_c_ is presented in the electronic supplementary material, figure S3.

The unloading portion of the pre-sliding friction response changed depending on the relaxation time, and the change was estimated by the model (unstable partial slip, figure 3) with the linear coupling parameter of *κ*. Please note that *κ* reported here was estimated by fitting the initial unloading portion (about 20 < *δ_N_*) because the experiments for long relaxation times exhibited nonlinear unloading after the initial unloading regime. *κ* showed a sharp decrease before *t*_relax_ = 50 s and gradually became steady towards *t*_relax_ = 200 s (figure 6*d*). The maximum value was 48.8 ± 15.65 at *t*_relax_ = 0 s, and the minimum value was 14.43 ± 6.81 at *t*_relax_ = 200 s.

**Figure 7.**
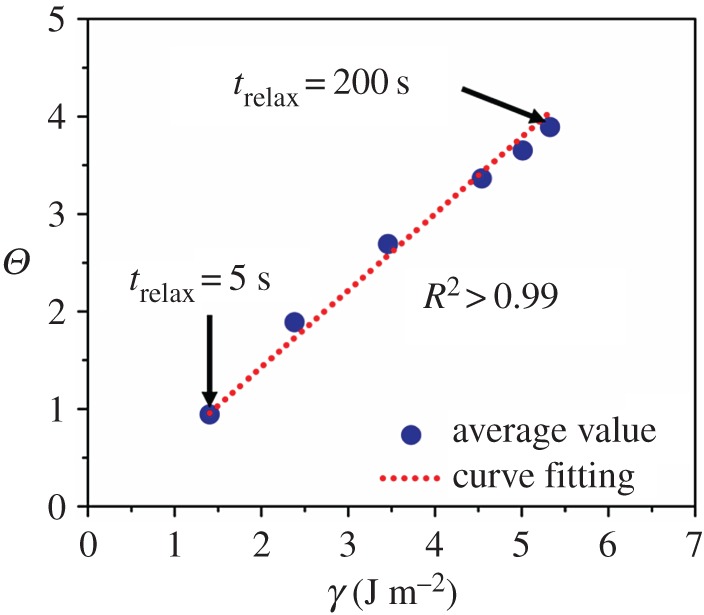
Correlation of the work of adhesion, *γ*, with the stick–slip contrast, *Θ* (*R*^2^ = 0.99). The graph shows that the resistance to the slip-based failure over the contact linearly increases with an increase in the work of adhesion.

The stick–slip contrast, *Θ*, showed a strong linear correlation with the work of adhesion, *γ*. Figure 7 presents *Θ* as a function of *γ*. The correlation between *γ* and *Θ* was quantitatively estimated by performing curve fitting with linear regression. As a result, the strong linear correlation was found to be *Θ* = −0.14 + 0.79*γ* with *R*^2^ > 0.99. Note that this curve fit has no applicability when *Θ* is less than about 1. *γ* and other parameters also showed linear correlations (*R*^2^ = 0.79 for *λ* and *R*^2^ = 0.84 for *κ*, as shown in the electronic supplementary material, figure S4), but they were not as strong as the linear correlation between *Θ* and *γ*.

## 4. Discussion

### 4.1. Relaxation-dependent adhesion of cartilage

The work of adhesion of cartilage, *γ*, was found to be relaxation dependent, and this was probably due to the combination of the poro-elasticity-driven suction effect, the formation of adhesive bridges within the contact area and the relaxation-driven geometric changes around the contact edges. The correlation between the work of adhesion and the relaxation time is consistent with the previous study on the hydrogel-on-glass contact [21]. The study reported that the adhesion is due to the poro-elasticity-driven suction effect under the indentation probe, arising from the imbalance of osmotic pressure. As hydrogel and cartilage have structural similarities (fibrous solid matrix swollen by fluids), it is expected that poro-elasticity-driven suction contributes to the adhesion of cartilage. The osmotic pressure is closely related to the degree of dehydration localized under the probe. The degree of localized dehydration can be linked to the relaxation response. Following this, the work of adhesion was predicted by substituting the normalized experimental relaxation curves in equation (2.2). Although the predicted adhesion showed a trend similar to the measured adhesion (equation (2.1)), there was a slight difference between them. A plausible reason for the slight difference might be the progressive formation of adhesive bridges between the spherical indenter and the cartilage constituents (e.g. collagen fibrils, hyaluronic acid and glycosaminoglycans (GAGs)) as reported in [20], resulting in the increase in the real contact area. Another possible mechanism linking adhesion and the time-dependent response of cartilage is changes in the shape of contact edges (crack) during relaxation (or creep). As shown by Greenwood & Johnson [33], loading and unloading rates of a contact will influence the shape of contact edges in a viscoelastic material. The relaxation-induced geometric changes will have a regularizing effect on the otherwise singular stresses at the contact edges, and thus increase the apparent work of adhesion measurements. Using this argument, a direct link between the time-dependent constitutive response and the work of adhesion can be constructed. The governing mechanisms of the adhesion of cartilage are beyond the scope of our work, but a similar dependence of the adhesion of cartilage on the relaxation time was successfully found on multiple locations on multiple samples (*Nn* = 15). The origins of the adhesion could be clearly determined by carefully controlling loading/unloading rates and using selectively modified samples (e.g. collagen digested, GAGs digested and dehydrated cartilage) and chemically treated probes. Finally, the work of adhesion measured here is compared with that calculated using a rigid punch solution (*v* = 0.25 assumed) [34] and published results [35]; the adhesion force during shearing under zero normal load was originally reported from the intercept of a linear regression line for the friction versus normal load data [35]. The calculated value is of the order of 0.1–1.6 J m^−2^, and is consistent with the range of our measurement (0.2–6.3 J m^−2^).

### 4.2. Pre-sliding friction response of cartilage with the slip-based failure model

The good agreement between the linear loading portion of the pre-sliding friction response and the fully adhered contact model provides a mechanics-based validation of the fully adhered contact and a confirmation of the relaxation-dependent shear modulus. In other words, the indenter was completely adhered to cartilage during the initial phase of tangential loading. Given that the cartilage is more compliant than the probe and transducer assembly, the tangential stiffness deduced from this initial adhered response can be attributed totally to deformations over cartilage. The shear moduli calculated from the tangential stiffness for *t*_relax_ ≤ 10 s cases are consistent with the range of previously reported values (mouse: 0.10–6.12 MPa [36]; bovine: 1.51 ± 0.37 MPa [37]; porcine: 0.36 MPa (converted from the elastic modulus by assuming *v* = 0.5) [38]). However, the shear moduli estimated for longer relaxation times are higher than the reported values. Although the higher values in our tests might be attributed to the sample species and test regions, the degree of water exudation in the cartilage–probe interface is probably the major reason. This is partially proved by the trend of the shear modulus (or *λ*) matching that of the relaxation curve and the consistency between moduli obtained from the adhesion and friction tests (see the electronic supplementary material). A previous study also reported that the relaxation and dehydration of cartilage can significantly increase its modulus [39]. Consequently, the relaxation-dependent shear modulus suggests that the modulus of cartilage increases along with its progressive dehydration induced by the localized pressure gradient. Furthermore, the correlation between the shear modulus and dehydration supports that poro-elasticity-driven suction occurs in the cartilage–probe interface as it is induced by localized dehydration, resulting in the imbalance of osmotic pressure.

The stick–slip contrast and the normalized radius of the critical stick area at failure are relaxation dependent. The stable partial slip of the pre-sliding friction response was well captured by the parameters, *Θ* and *χ*_c_, of the model. The increasing trend of *Θ* with relaxation time shows that the resistance to the slip-based failure over the contact exponentially increases with the relaxation time. This indicates that a larger tangential force is required to initiate the peeling of the fully adhered contact area as the relaxation time increases. The increases in *Θ* and *χ*_c_ suggest that the fully adhered (stick) contact formed in the linear loading portion is better maintained with increases in the stick–slip contrast and the relaxation time, indicating less of an annular slip area (i.e. fracture process zone). As *χ*_c_ ranged between 0.8 and 0.98, the small-scale failure assumption is valid for the set of experiments analysed.

The strong linear correlation between *Θ* and *γ* shows that the relaxation-dependent work of adhesion of cartilage dominantly governs its resistance to tangential loading and slip-based failure. The higher work of adhesion could mean that the poro-elasticity-driven suction and adhesive bridges become stronger over time. Therefore, a higher tangential load is required to break the adhesion (stick) formed by them. Such increased adhesion could induce severe damage on cartilage as observed in [20]. Other studies on the contact interfaces of soft materials support our findings. A hydrogel–glass interface showed that the poro-elasticity-driven suction effect resists the lateral sliding of a probe [21]. Lubricated elastomer contacts showed that the break-loose friction increases with the time of stationary contact because the real contact area increases with the progression of fluid squeeze-out and de-wetting [40]. In addition, earlier studies showed that the contact interfaces between identical soft materials (hydrogel-on-hydrogel [41] and cartilage-on-cartilage [20]) exhibit a similar stick–slip trend simulated by the slip-based failure studied here. Therefore, the slip-based failure model has the potential to explain the mechanics of cohesive contacts between soft materials.

The linear correlation between the maximum tangential load and adhesion is in line with the initially adhered contact and adhesion-controlled failure assumptions employed in the model. Accordingly, the critical energy release rates in our pre-sliding tests should scale as $G_{c}∼\Theta^{2}/E^{*}$. Specifically, in a mode II loading, $G_{c}∼(K_{IIc}^{2}/E^{*})={\left(\Theta Q_{s}\right)}^{2}/E^{*}a^{3}$, and because the contact radius and sliding friction force in our tests were approximately the same for all cases, $G_{c}∼\Theta^{2}/E^{*}$. As extracted from the contact stiffness, the equivalent shear modulus increases nearly 2.1-fold from *t*_relax_ = 5 s to *t*_relax_ = 200 s in our experiments. Assuming similar variation occurs in the equivalent Young's modulus of the contact, E*, the energy release rate $G_{c}∼\Theta^{2}/E^{*}$ is expected to increase eightfold from *t*_relax_ = 5 s to *t*_relax_ = 200 s. This increase is not too different from the fourfold increase in the work of adhesion. Note that mode-mixing and dissipative interactions during the pre-sliding tests could contribute to the apparent energy release rates estimated, and lead to this slight mismatch between the increases in energy release rates and the work of adhesion [24,42,43]. The critical stick areas at the onset of slip-based failure are estimated to be large in comparison with the peeled area (see electronic supplementary material, figure S3). Therefore, brittle failure with negligible mode-mixing is a plausible assumption applicable to the experiments [24]. Dissipative interactions between the probe and cartilage samples are expected to be more prominent in pre-sliding than in adhesion tests. This is because loading and unloading rates during adhesion tests are much higher than 2.5 s sliding durations in pre-sliding tests. Within longer durations, larger portions of the mechanical work will be expended to dissipative processes such as relaxation and diffusion in the tissue. Therefore, the apparent energy release rates estimated will be higher than the work of adhesion. This trend is in line with the previous results of peeling tests from Maugis & Barquins [43]; polyurethane strips were peeled from glass at different peel angles, and they reported that measured energy release rates were higher than the actual work of adhesion. The contribution of dissipative interactions can be independently verified by additional experiments with a range of sliding velocities. However, increasing sliding velocities could trigger rehydration and hydrodynamic interactions at the probe–cartilage interfaces [28,29]. The authors are currently exploring options to decouple those mechanisms, and their effects on the pre-sliding response. Nevertheless, the strong linear correlation between *Θ* and *γ* ($\approx G_{c}$) found in this paper suggests the possibility of predicting the relaxation-dependent work of adhesion from the pre-sliding friction response of cartilage-like hydrated materials, and provides the physical basis and predictive capability to *in vivo* diagnostic techniques for biomedical interfaces (e.g. shear wave elastography [44]).

The decrease in the linear scaling parameter, *κ*, with the relaxation time shows that the annular slip area extends faster towards the centre of the contact in the relaxed state. Although the unstable partial slip model with *κ* provided valuable insight into the extension rate of the slip area in the initial unloading portions (*δ_N_* < 20), it failed to predict the extension behaviour in the latter unloading portions from relatively long relaxation times (figure 5*b*). This failure suggests that higher stick–slip contrast induces a nonlinear relationship between the relative tangential velocity of the contacting interfaces and the extension rate of the annular slip area. Note that Brochard-Wyart & de Gennes [26] also used a linear scaling between the imposed displacement rates and crack-tip velocities as $\kappa ∼\varepsilon_{0}$, where *ϵ*_0_ is the threshold strain beyond which slip-based failure over the contact commences. Moreover, they showed that crack-tip velocities remained relatively constant throughout the peeling process. Even under those crude assumptions, their model successfully captured experimentally observed stick–slip transitions in PDMS-on-glass contacts [26,32]. Despite their successful application of the linear scaling, our experiments show that more sophisticated unloading models accounting for nonlinearities such as ploughing, self-healing and collective diffusion should be employed (see [45,46] for the physical basis of those nonlinearities).

Reale & Dunn [21] have studied the friction response of hydrogels experimentally, and proposed a simple model for friction of soft hydrated materials, and, therefore, their model is worth comparing with our work. The model of Reale and Dunn assumes rigid traversing of a probe over relaxed (dehydrated) and unrelaxed (hydrated) parts of soft hydrated materials, and simple areal mixing rules to estimate the friction force during unloading. Their model could not explain the pre-sliding friction results of cartilage; it overestimated the peak friction coefficients and the lateral displacement where the coefficient of friction converges (see the electronic supplementary material). The slip-based failure model, on the other hand, could explain the onset of slip and subsequent debonding better. Notwithstanding the promising fits to the experiments, the slip-based model has deficiencies in representing the decohesion (unloading) regime, which could be resolved with *in situ* experiments and nonlinear peeling kinematics as discussed above.

As cartilage contains a large amount of water, it is necessary to consider if the hydrodynamic effects and adhesion played a role during the application of a tangential displacement. When hydrodynamic effects are present at the interface, the viscous energy dissipation, energy release rate and the friction coefficient increase with increasing sliding velocity. The velocity employed in the friction tests was sufficiently low not to interfere with the rehydration process induced by the pressure gradient. For instance, cartilage requires sliding velocities of the order of tens of millimetres per second to restore hydration after water exudation [28]. Therefore, it is reasonable to consider that hydrodynamic effects did not significantly influence the pre-sliding friction responses reported here. In addition, the duration of each pre-sliding test was 2.5 s. Therefore, we do not anticipate any significant change in the contact relaxation and adhesion during pre-sliding tests. Substantial changes in the work of adhesion, which significantly influenced the pre-sliding friction response, were due to significant relaxation that occurred before the application of the tangential displacement.

### 4.3. Assumptions and limitations of the slip-based failure model

The crude assumptions and limitations of the slip-based failure model are worth revisiting. This model adopts brittle fracture initiation and assumes a small-scale process zone size. Various regularization methods with mixed-mode debonding, frictional slip with Coulomb law and plastic yielding at the interface are offered to limit infinite shear tractions at the edge of the sticking contact [47–52]. For instance, mixed-mode debonding at the interfaces between inclusions in soft matrix, fibre-reinforced composites and jointed surfaces is modelled accurately with coupled cohesion-friction models [52]. In this model, the interface obeys the cohesive constitutive law with a smooth traction–separation relation [53]. Once the tangential traction reaches its maximum, the debonding commences and frictional tractions occur between the debonded parts of the interface. After that point, the cohesive strength diminishes, while the regions under frictional slip increase until the onset of gross sliding. Although this cohesive zone model promises similar results to the slip-based failure model, the physical basis deteriorates because of the large number of input parameters required. Ongoing work by the authors considers analogies between these models, and, thus, attempts to link mechanics-based processes at the interface to the input parameters of the cohesive models. Such a model would employ observable parameters, possess the physical basis and enable relaxation of the small-scale process zone assumption.

The model also ignores the regularizing effects of crack-tip blunting and cavitation on the interfacial tractions, and therefore it is worthwhile considering their effects. As discussed thoroughly by others [54,55], an elasto-adhesive length determines the significance of blunting and cavitation of soft materials in comparison with the smallest of the other geometric features in a given failure case. The length can be estimated as $l_{EA}=(G_{c}/E)\approx (\gamma /E)$. For the probe-based tangential loading, the contact radius can serve as the smallest dimension. The elasto-adhesive lengths can be estimated by using the work of adhesion and the shear modulus reported in the results section. At *t*_relax_ = 200 s, the work of adhesion is 5.33 J m^−2^, and the elastic modulus, *E*, is estimated to be 33.20 ± 11.58 MPa (*E* = 2*G*(1 + *v*), *G* = 13.28 ± 4.63 MPa, and *v* = 0.25); this is consistent with *E* calculated using the load–displacement curves measured in the adhesion tests (see the electronic supplementary material). With those values, *l*_EA_ at *t*_relax_ = 200 s is estimated as 160.54 nm for the cartilage (89.03 nm for *t*_relax_ = 5 s). As the contact radius in our experiments (≈250 µm) is significantly greater than the estimated *l*_EA_, the interfacial failure in our experiments is expected to exhibit brittle-like behaviour with negligible blunting and cavitation.

## 5. Conclusion

This study experimentally demonstrated the relaxation-dependent adhesion of cartilage and its effect on the pre-sliding (static) friction response of cartilage. In addition, the slip-based failure model provided the mechanistic explanation of the pre-sliding friction behaviour and the stick–slip response of cartilage. The work of adhesion of cartilage increased with a progression of relaxation. The increase in the work of adhesion linearly increased the resistance of the slip-based interface failure, resembling fast crack growth. Also, as the work of adhesion increased, the size of the critical adhered (stick) area at failure became larger, and the extension rate of the annular slip area towards its centre (crack growth) increased in the initial unloading portion. These findings fill the gap in our knowledge about the pre-sliding (static) friction response of cartilage, leading to a better understanding of the stick-induced damage on its surface. The quantitative interpretation of the friction response with the mechanics-based model can be a powerful method when the contact areas between soft-hydrated materials (e.g. cartilage) are not observable. To further our research, we are planning to investigate the effect of the tangential displacement rate on the pre-sliding friction response. In addition, further work will include the improvement of the unstable partial slip model to capture the nonlinear behaviour observed in the late stage of the unloading portion.

## Supplementary Material

Supplementary material

## Data accessibility

Datasets are available from the Dryad Digital Repository (https://doi.org/10.5061/dryad.v0108) [56].
