# Supplementary material for: Effect of relaxation-dependent adhesion on pre-sliding response of cartilage
Source: R Soc Open Sci. 2018 May 23;5(5):172051. doi: 10.1098/rsos.172051 (PMC5990745; doi:10.1098/rsos.172051)
Supplement: Supplementary material [file rsos172051supp1.docx]

Supplementary materials:

Effect of Relaxation-Dependent Adhesion on Pre-Sliding Response of Cartilage

Guebum Han^a,^ * and Melih Eriten^a^ (*Corresponding author)

^a^Department of Mechanical Engineering, University of Wisconsin-Madison, Madison, WI 53706, USA


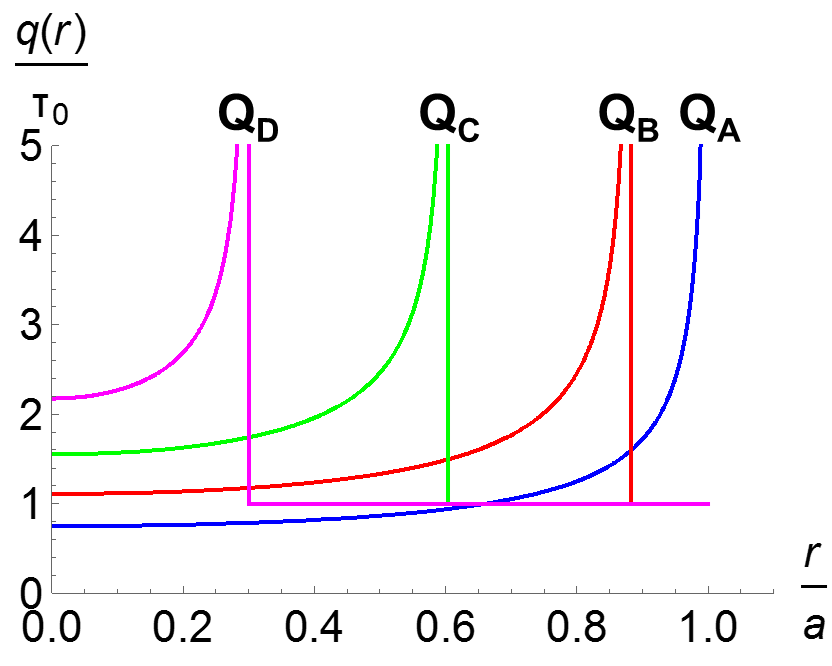


**Figure S1. Shear traction distribution over the contact radius at points Q_A_, Q_B_, Q_C_, and Q_D_ (refer to the colored version for matching of points and traction profiles).**


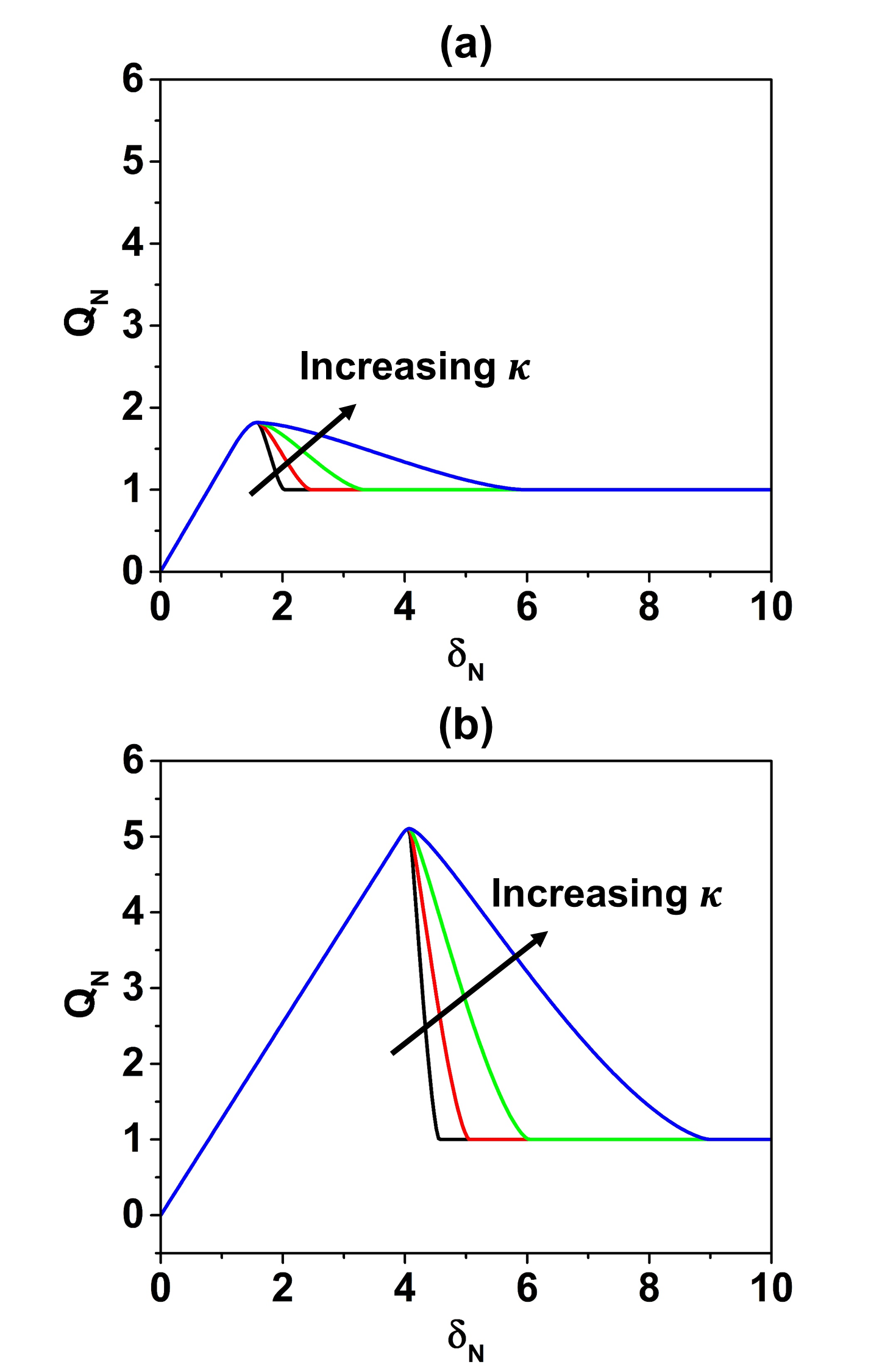


**Figure S2. Representative of slip-based failure model for a) Θ=1.5 and b) Θ=5. The family of curves indicates the influence of scaling parameter κ on the unloading response (κ=0.5,1,2,5).**


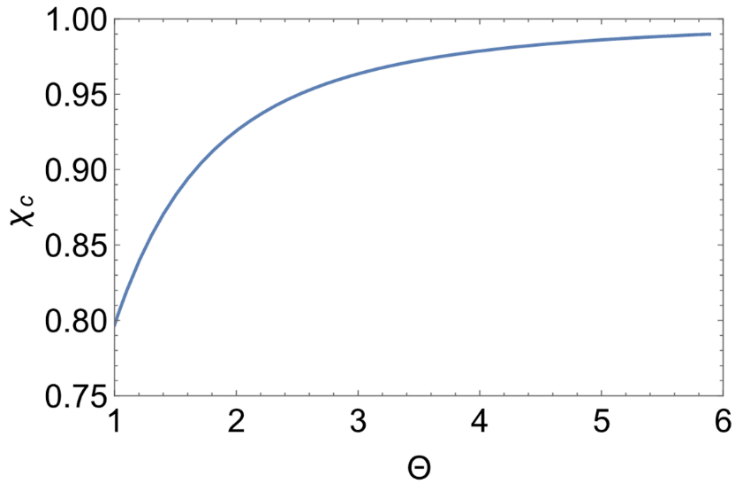


**Figure S3. Critical stick radius** $\boldsymbol{\chi}_{\boldsymbol{c}}$ **as a function of** $\boldsymbol{\Theta=}\frac{\boldsymbol{Q}_{\boldsymbol{A}}}{\boldsymbol{Q}_{\boldsymbol{S}}}$**.** $\boldsymbol{\chi}_{\boldsymbol{c}}$ **was determined by using Eq. (S20).**

*
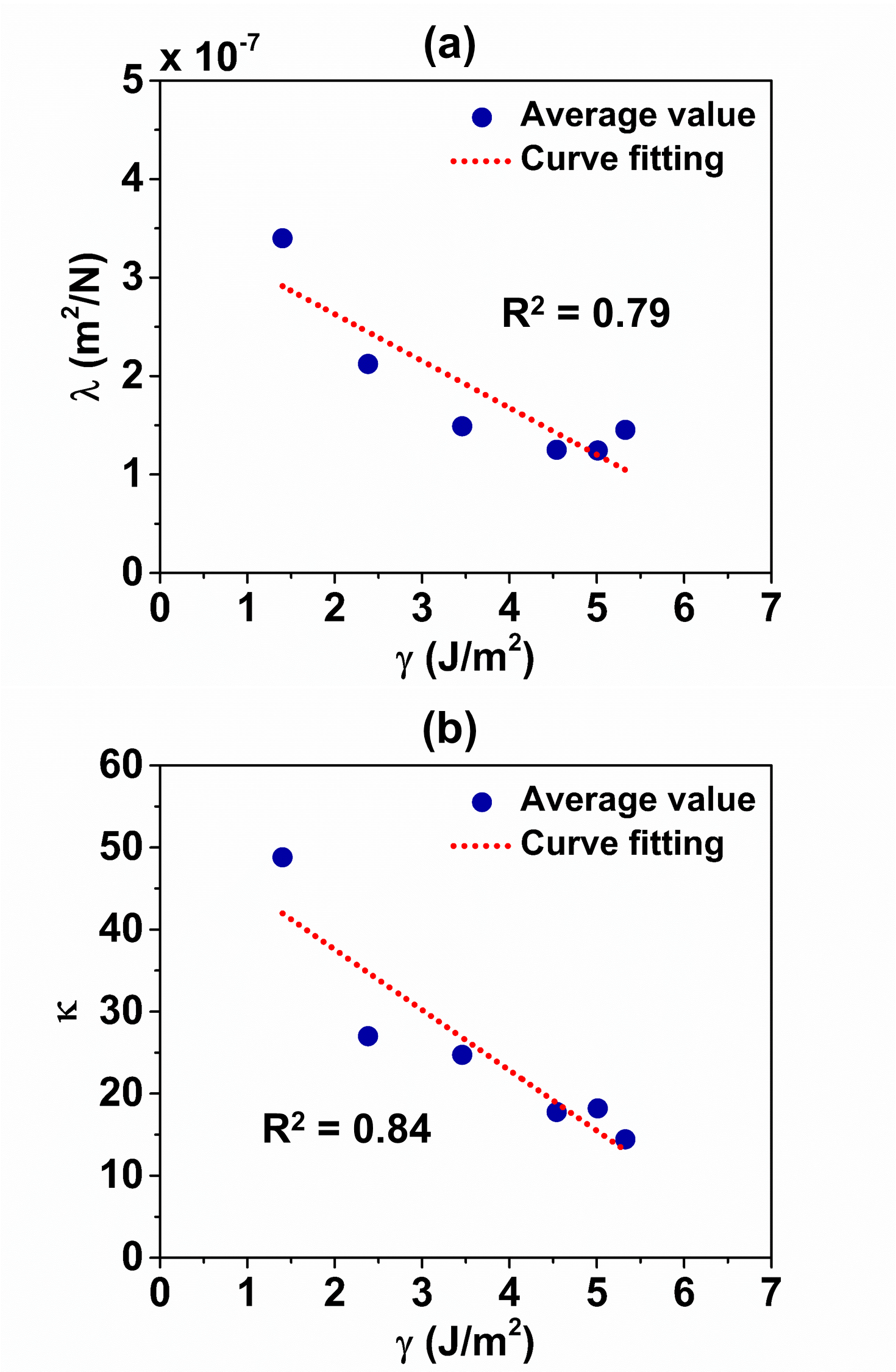
*

**Figure S4. Correlations of work of adhesion with (a)** $\boldsymbol{\lambda}$ **and (b)** $\boldsymbol{\kappa}$**.**

# Details of the Slip-Based Failure Model

Note that some of the equations below overlap with the equations in the paper. They were not removed on purpose because the current format makes it easier to follow.

# Fully-Adhered Contact (Q_0_ – Q_A_)

A tangential traction, $q\left( r \right)$, in a fully adhered contact with a contact radius of $a$ is given by [1]:

|  | $q\left( r \right)=\frac{Q}{2\pi a^{2}}\left( 1-\left( \frac{r}{a} \right)^{2} \right)^{-1/2}$ | *(S1)* |
| --- | --- | --- |

, where $r$ is the radial coordinate from the center of the contact area and $Q$ is the tangential force. $q\left( r \right)$ vanishes for $r>a$. Associated stress intensity factor in shear can be estimated as

|  | $K_{II}=\frac{Q}{\pi\left( 2a \right)^{3/2}}$ | *(S2)* |
| --- | --- | --- |

Note that the stress intensity factor given in Eq. (S2) is defined as $\lim_{r\to a} q\left( r \right)\sim\frac{K_{II}}{\sqrt{a-r}}$. The tangential displacement, $\delta$, is linked to $Q$ linearly as:

|  | $\delta=\frac{\lambda Q}{8a}; \lambda=\frac{2-\nu_{1}}{G_{1}}+\frac{2-\nu_{2}}{G_{2}}$ | *(S3)* |
| --- | --- | --- |

, where $\nu_{i}$ and $G_{i}$are the Poisson’s ratio and shear modulus of $i^{th}$contacting material. The normalized forms of the tangential force, $Q_{N}$, and the tangential displacement, $\delta_{N}$, are defined as follows:

| $Q_{N}=\frac{Q}{Q_{S}}= \frac{Q}{\tau_{0}\pi a^{2}}$ | *(S4)* |
| --- | --- |
| $\delta_{N}=\frac{2\delta}{a\lambda\tau_{0}}$ | *(S5)* |

where $Q_{S}=\tau_{0}\pi a^{2}$ is the tangential force when the whole of the contact area slips (gross sliding). Then the relationship between $Q_{N}$ and $\delta_{N}$ in the fully adhered contact is established by substituting Eq. (S4) and Eq. (S5) into Eq. (S3) as follows:

| $Q_{N}=\frac{4}{\pi}\delta_{N}$ | *(S6)* |
| --- | --- |

# Stable Partial Slip (Q_A_ – Q_B_)

At the onset of stable partial slip ($Q=Q_{A}$), the stress intensity factor attains the critical value of fracture toughness, $K_{IIc}$, as follows [2]:

|  | $K_{II}=\frac{Q_{A}}{\pi\left( 2a \right)^{3/2}} =K_{IIc}, or Q_{A}=\pi\left( 2a \right)^{3/2}K_{IIc}$ | *(S7)* |
| --- | --- | --- |

A subsequent increase in tangential force leads to the shrinkage of the fully-adhered contact area from a radius of $a$ to $c$ (Figure 2a). As a result, the annular debonded area ($c<r<a)$ is generated, and it experiences frictional slip with $\tau_{0}$. The slip-based failure model [2,3] superposes the solutions of two sub-problems associated with the fully-adhered and annular slip contact areas. The two sub-problems can be solved for the tangential tractions, displacements and forces with boundary conditions. The 1^st^ sub-problem is to find a tangential force of a fully adhered contact with a radius of $c$. The solution to this sub-problem yields expressions identical to Eqs. (S1) and (S3) with a contact radius of $c$ instead of $a$:

|  | $q_{1}\left( r \right)=\frac{Q_{1}}{2\pi c^{2}}\left( 1-\left( \frac{r}{c} \right)^{2} \right)^{-1/2}$ | *(S8)* |
| --- | --- | --- |

|  | $\delta_{1}=\frac{\lambda Q_{1}}{8c}; \lambda=\frac{2-\nu_{1}}{G_{1}}+\frac{2-\nu_{2}}{G_{2}}$ | *(S9)* |
| --- | --- | --- |

In this sub-problem, the contact is assumed to remain in the critical failure state as follows:

| $Q_{1}=\pi\left( 2c \right)^{3/2}K_{IIc}$ | *(S10)* |
| --- | --- |

Note that the influence of the 1^st^ sub-problem on the total solution decreases with the shrinkage of the fully-adhered contact area. The 2^nd^ sub-problem is to find a tangential traction satisfying a constant displacement in the fully-adhered region, $\delta_{2}$, and a constant residual traction in the annular slip region, $\tau_{0}$ (i.e., fracture process zone). The tangential traction satisfies those conditions as follows:

|  | $q_{2}\left( r \right)=\left\{ \begin{matrix} \frac{\tau_{0}}{\pi}\cos^{-1} \frac{2c^{2}-a^{2}-r^{2}}{a^{2}-r^{2}} & r\leq c \\ \tau_{0} & c<r\leq a \end{matrix} \right.$ | *(S11)* |
| --- | --- | --- |

The corresponding tangential force and displacement can be found as follows:

|  | $Q_{2}=\int_{0}^{a} 2\pi q_{2}\left( r \right)rdr=2\tau_{0}a^{2}\left( \cos^{-1} \frac{c}{a}+\frac{c}{a}\sqrt{1-\left( \frac{c}{a} \right)^{2}} \right)$ | *(S12)* |
| --- | --- | --- |

|  | $\delta_{2}=\frac{\lambda\tau_{0}a}{2}\sqrt{1-\left( \frac{c}{a} \right)^{2}}$ | *(S13)* |
| --- | --- | --- |

Consequently, the tangential traction, tangential force and tangential displacement during the stable partial slip contact (i.e., growth of the process zone) are obtained by superposing those two solutions as follows:

|  | $q\left( r \right)=\left\{ \begin{matrix} \frac{{\sqrt{2}K}_{IIc}}{\sqrt{c}}\left( 1-\left( \frac{r}{c} \right)^{2} \right)^{-1/2}+\frac{\tau_{0}}{\pi}\cos^{-1} \frac{2c^{2}-a^{2}-r^{2}}{a^{2}-r^{2}} & r\leq c \\ \tau_{0} & c<r\leq a \end{matrix} \right.$ | *(S14)* |
| --- | --- | --- |
|  | $Q=\pi\left( 2c \right)^{3/2}K_{IIc}+2\tau_{0}a^{2}\left( \cos^{-1} \frac{c}{a}+\frac{c}{a}\sqrt{1-\left( \frac{c}{a} \right)^{2}} \right)$ | *(S15)* |
|  | $\delta=\frac{\pi\lambda K_{IIc}\sqrt{c}}{2\sqrt{2}}+\frac{\lambda\tau_{0}a}{2}\sqrt{1-\left( \frac{c}{a} \right)^{2}}$ | *(S16)* |

Eqs. (S14-S16) are normalized as follows:

| $q_{N}\left( r \right)=\frac{q\left( r \right)}{\tau_{0}}=\left\{ \begin{matrix} \frac{\Theta}{2\sqrt{\chi\left( \chi^{2}-\rho^{2} \right)}}+\frac{1}{\pi}\cos^{-1} \frac{2\chi^{2}-\rho^{2}-1}{1-\rho^{2}} & \rho\leq\chi\\ 1 & \chi<\rho\leq1 \end{matrix} \right.$ | *(S17)* |
| --- | --- |
| $Q_{N}=\frac{Q}{Q_{S}}=\frac{Q}{\tau_{0}\pi a^{2}}=\Theta\chi^{3/2}+\frac{2}{\pi}(\chi\sqrt{1-\chi^{2}}+\cos^{-1}\chi)$ | *(S18)* |
| $\delta_{N}=\frac{2\delta}{\lambda\tau_{0}a}=\frac{\pi}{4}\Theta\sqrt{\chi}+\sqrt{1-\chi^{2}}$ | *(S19)* |

where $\Theta=\frac{Q_{A}}{Q_{S}}=\frac{2^{\frac{3}{2}}K_{IIc}}{\tau_{0}a^{1/2}}$ is the ratio of the critical to sliding tangential forces (called as stick-slip contrast), $\chi=\frac{c}{a}$ is the normalized radius of the fully-adhered (stick) contact area, and $\rho=\frac{r}{a}$ is the normalized radial coordinate, respectively.

Next, we can determine the normalized critical stick radius$\chi_{c}=\frac{c_{c}}{a}$ satisfying $\frac{dQ_{N}}{d\chi}=0$ by using Eq. (S18), and obtain the following equation for a given $\Theta=\frac{Q_{A}}{Q_{S}}$

|  | $\Theta=\frac{8\chi_{c}^{3/2}}{3\pi\sqrt{1-\chi_{c}^{2}}}$ | *(S20)* |
| --- | --- | --- |

At this critical stick radius, the energy release rate at the highly stressed edge of the fully-adhered (stick) area reaches a critical value, and the annual slip area starts to grow toward its center.

# Unstable Partial Slip (Q_B_ –Before Gross Sliding)

At the maximum tangential force, $Q_{B}$, the energy release at the edge of the fully-adhered area exceeds the frictional dissipation, and the annual frictional slip (debonded) area grows quickly and unstably as illustrated in Figure 2a (i.e., propagation of crack). Beyond this point, a tangential force decreases with a further increase in a tangential displacement, and thus unloading occurs (Figure 2b). In an experiment, unstable unloading will be suppressed by constraints between the contacting surfaces (e.g., control of relative displacement between sample and probe [4]). To meet the constraints in a setup with displacement control, the relative tangential velocity of the contacting interfaces, $\dot{\delta}$, is assumed to scale linearly with the extension rate of the annual frictional slip zone,$\dot{\chi}_{un}$. Then its normalized form, $\dot{\delta_{N}}$, can be expressed as follows:

| $\dot{\delta}_{N}=-\kappa\dot{\chi}_{un}$ | *(S21)* |
| --- | --- |

where $\kappa$ is a scaling constant. The normalized tangential displacement during the unstable partial slip, $\delta_{N}$, can be eventually expressed as follows:

| $\delta_{N}=\delta_{N}^{B}+\kappa\left( \chi_{c}-\chi_{un} \right)$ | *(S22)* |
| --- | --- |

where $\delta_{N}^{B}$ is the normalized tangential displacement at $Q=Q_{B}$, $\chi_{c}$ is the normalized radius of the fully-adhered (stick) contact area at $Q=Q_{B}$, and $\chi_{un}=\frac{C_{un}}{a}$ is the normalized radius of the stick contact area during unloading. The normalized tangential force, $Q_{N}$, is assumed to attain the same value corresponding to the stable partial slip (Eq. (S18)) with $\chi_{un}$ instead of $\chi$. Note that kinematic constraints localized at the interface might exhibit nonlinear correlation to the crack tip velocities. The linear correlation offered here should be treated as a first order approximation.

# Calculation of Elastic Modulus with Load-Displacement Curves from Adhesion Tests

Elastic moduli were calculated with the loading and unloading portions of the load-displacement curves from the adhesion tests, and they were consistent with the elastic moduli converted from the shear moduli, obtained from the friction tests. The elastic modulus from the loading curves was obtained by fitting the Hertz contact model as follows (Figure S5) [5]:

| $F=\frac{4}{3}{\frac{E}{1-v^{2}}R}^{\frac{1}{2}}d^{\frac{3}{2}}$ | *(S23)* |
| --- | --- |

where F is the normal load, E is the elastic modulus, ν is Poisson’s ratio, R is the indenter radius (= 1mm), and d is the normal displacement. The tip was assumed to be rigid. ν = 0.5 was assumed because no volume change was expected during the application of instantaneous load [6]. The loading curves used were from the adhesion tests without relaxation (15 curves). The elastic modulus was calculated to be 13.26±1.51 MPa, and it can represent the modulus of hydrated cartilage because the loading curves were not affected by the significant exudation of fluid. The elastic modulus (13.26±1.51 MPa) from the loading curves was similar to that (18.88±10.22 MPa) from the pre-sliding friction tests at $t_{relax}=5 s,$ converted from the shear modulus by assuming ν = 0.5.

The elastic modulus of dehydrated cartilage was calculated from the unloading curves by using the Oliver-Pharr method as follows [7]:

| $\left( \frac{1-v^{2}}{E} \right)^{-1}=\frac{\sqrt{\pi}}{2}\frac{s}{\sqrt{A}}$ | *(S24)* |
| --- | --- |

where S is the elastic stiffness from the slope of the unloading curve (Figure S6) and A is the contact area at the maximum penetration depth (A = $\pi a^{2}$ = $\pi$Rd = $\pi$(1 mm)(60 μm)). The rigid tip and ν = 0.25 were assumed. The unloading curves used were from the adhesion tests at $t_{relax}=200 s$ (15 curves). The elastic modulus was calculated to be 32.80±6.29 MPa, and it can represent the modulus of dehydrated cartilage. The elastic modulus (32.80±6.29 MPa) from the unloading curves matched with that (33.20±11.58 MPa) from the pre-sliding friction tests with $t_{relax}=200 s$, converted from the shear modulus by assuming ν = 0.25.


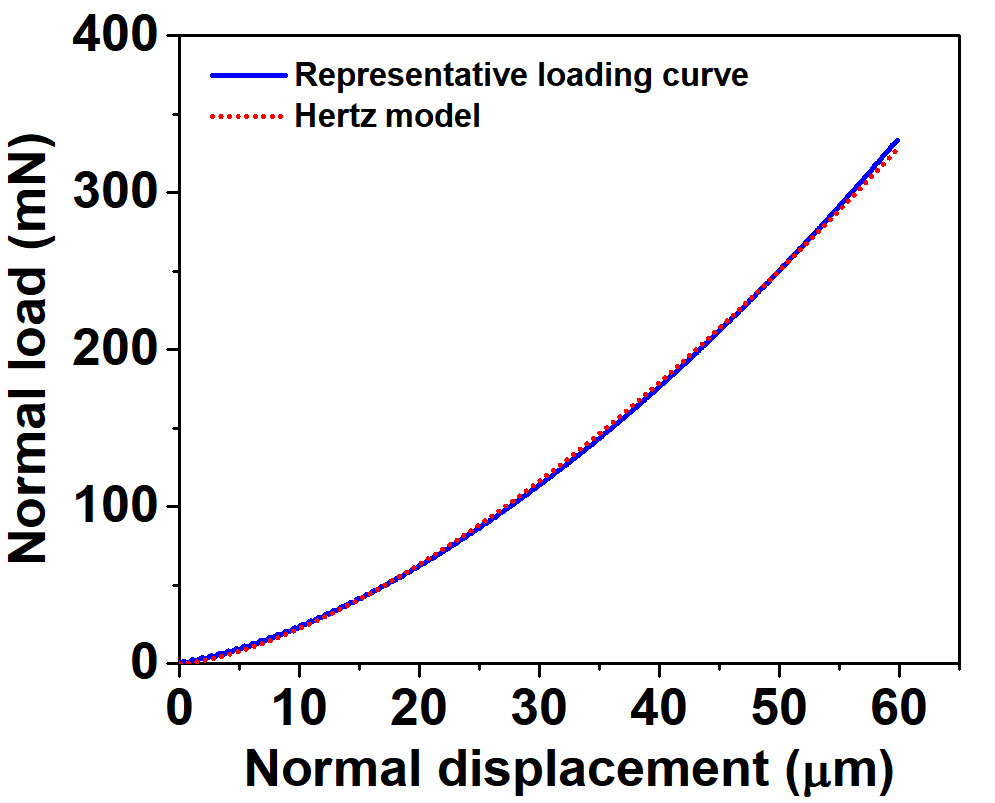


**Figure S5. Representative of loading curves with Hertz model. Elastic modulus was determined by fitting the Hertz model to the loading curves. The R-squared values for all of the fits were 0.995±0.005.**


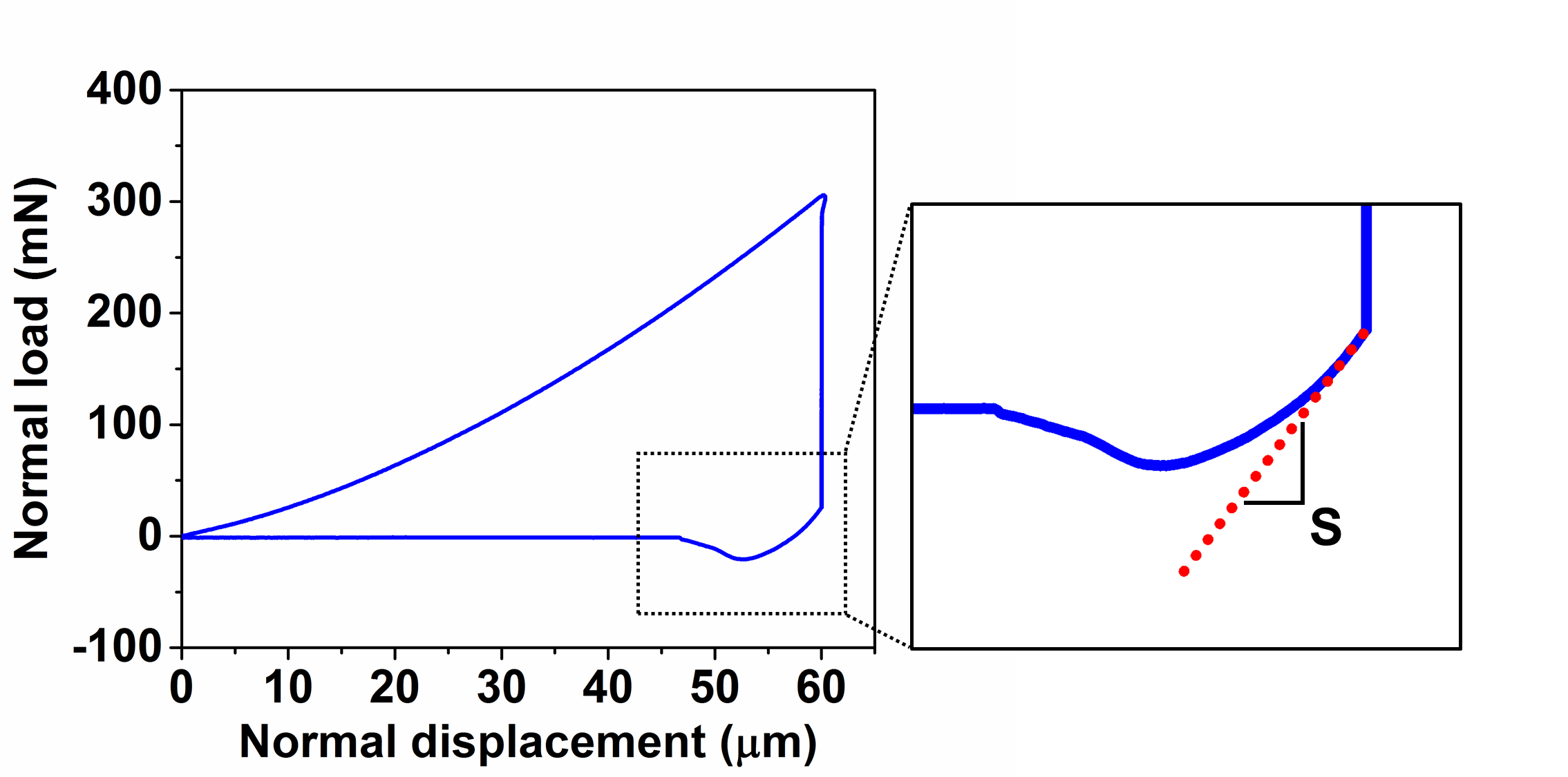


**Figure S6. Representative of unloading curves with elastic stiffness.**

# Application of Static Friction Model Based on Mixture Approach

The static friction model of Reale and Dunn (RD) was applied to the pre-sliding friction results of cartilage at different relaxation times. The RD model only covers the unloading regime of pre-sliding friction response, and it is given in their paper as follows [8]:

| $F_{f}=\left( \frac{2a^{2}}{l} \right)[\frac{\gamma_{h}\pi}{2}+\left( \gamma_{d}-\gamma_{h} \right)\left( \cos^{-1} b-\sqrt{b^{2}-b^{4}} \right)]$ | *(S25)* |
| --- | --- |

where $F_{f}$ is the friction force, a is the contact radius, $l$ is the characteristic length scale, $\gamma_{h}$ is the surface energy (work of adhesion) of a hydrated interface, $\gamma_{d}$ is the surface energy (work of adhesion) of a dehydrated interface, and b is x/2a (x is the lateral displacement). It can be expressed as coefficient of friction, $\mu$:

| $\mu=\frac{F_{f}}{F_{n}}=\left( \frac{2a^{2}\gamma_{h}}{lF_{n}} \right)[\frac{\pi}{2}+\left( \frac{\gamma_{d}}{\gamma_{h}}-1 \right)\left( \cos^{-1} b-\sqrt{b^{2}-b^{4}} \right)]$ | *(S26)* |
| --- | --- |

Since $l$ is the fitting parameter, $\mu$ can be simply written as follow:

| $\mu=\frac{F_{f}}{F_{n}}=C [\frac{\pi}{2}+\left( \frac{\gamma_{d}}{\gamma_{h}}-1 \right)\left( \cos^{-1} b-\sqrt{b^{2}-b^{4}} \right)]$ | *(S27)* |
| --- | --- |

where $C=\frac{2a^{2}\gamma_{h}}{lF_{n}}$ is the effective fitting parameter.

The RD model (Eq. (S27)) was first fitted to the representative results of the friction tests in cartilage given in Figure S7a by determining *C* based on the steady state (kinetic) friction coefficient, as done in their paper [8]. The model overestimated the peak friction coefficients (about 5 times) and the lateral displacement at the steady sliding (Figure S7b). The experimental results showed that the coefficient of friction converges after sliding about half of the contact diameter, while the RD model predicted that it converges after sliding about the contact diameter (=2a).

Since the RD model extremely overestimated the coefficient of friction with the above fitting procedure, it was fitted to the experimental data by selecting *C* based on the peak friction coefficients. Although the model matched the peak friction coefficients owing to the proper selection of *C*, it still overestimated the point where the coefficient of friction converges (after sliding about the contact diameter) (Figure S7c).


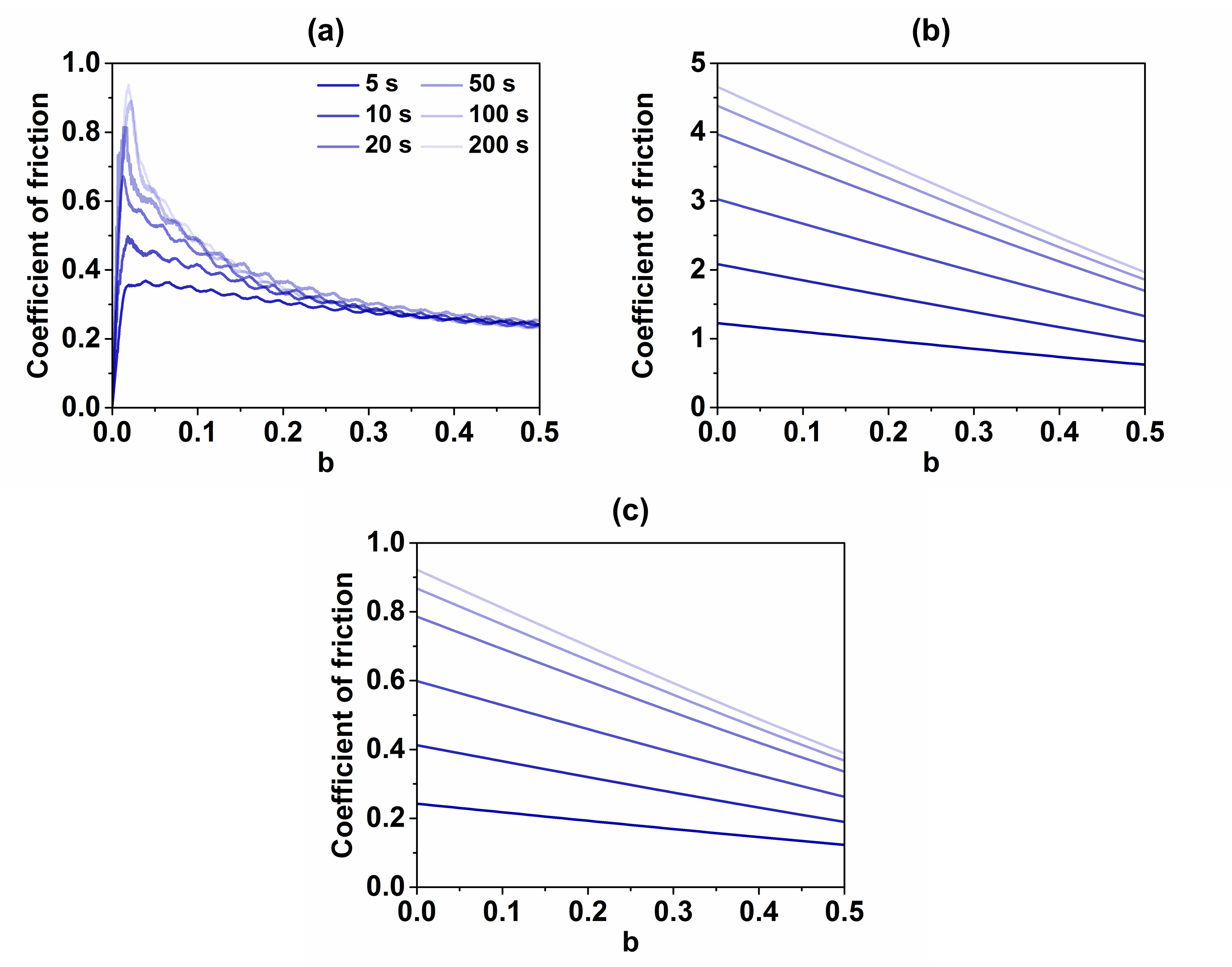


**Figure S7. (a) Representative results of friction tests in cartilage; (b and c) prediction of results with Reale and Dunn model. (b) and (c) were obtained by fitting their model to the experimental results based on the steady state (kinetic) friction coefficient and the peak friction coefficients, respectively.**

# References

1. Mindlin RD. 1949 Compliance of Elastic bodies in contact. *J. Appl. Mech.* **16**, 259–268.

2. Savkoor AR. 1987 Dry Adhesive Friction of Elastomers: A study of the fundamental mechanical aspects.

3. Singer IL, Pollock H. 1992 *Fundamentals of Friction: Macroscopic and Microscopic Processes*. Springer Science & Business Media.

4. Calomino AM, Brewer DN. 1992 Controlled Crack Growth Specimen for Brittle Systems. *J. Am. Ceram. Soc.* **75**, 206–208. (doi:10.1111/j.1151-2916.1992.tb05467.x)

5. Hertz H. 1881 On the contact of elastic solids. *J. Reine Angew. Math.* **92**, 156–171.

6. Oyen ML. 2008 Poroelastic nanoindentation responses of hydrated bone. *J. Mater. Res.* **23**, 1307–1314. (doi:10.1557/JMR.2008.0156)

7. Oliver WC, Pharr GM. 1992 An improved technique for determining hardness and elastic modulus using load and displacement sensing indentation experiments. *J. Mater. Res.* **7**, 1564–1583. (doi:10.1557/JMR.1992.1564)

8. Reale ER, Dunn AC. 2017 Poroelasticity-driven lubrication in hydrogel interfaces. *Soft Matter* **13**, 428–435. (doi:10.1039/C6SM02111E)
